# Uncontrolled Epistaxis Secondary to Traumatic Pseudoaneurysm of the Maxillary Artery

**DOI:** 10.1155/2011/347671

**Published:** 2011-12-06

**Authors:** Eelam Adil, Dhave Setabutr, Michele M. Carr

**Affiliations:** Division of Otolaryngology-Head and Neck Surgery, Pennsylvania State University, 500 University Drive Hershey, PA 17036, USA

## Abstract

We describe a rare case of traumatic pseudoaneurysm of the maxillary artery following a fall. The patient presented with epistaxis that could not be controlled with anterior and posterior nasal packing. She was urgently taken to the angiography suite for evaluation and ultimately underwent embolization of a left maxillary artery pseudoaneurysm with 500–700 micron Contour PVA followed by coiling with two 3 mm Tornado coils. Bleeding subsided after embolization, and the patient suffered no neurologic sequelae.

## 1. Introduction

Patients with intractable epistaxis commonly present to the emergency room for evaluation. Bleeding can be controlled with conservative measures including medications such as oxymetazoline, pressure, and/or nasal packing. Intractable epistaxis may require surgical intervention through a Caldwell-Luc approach to the sphenopalatine artery. Embolization has emerged as a less invasive means of controlling intractable bleeding. We present an interesting case of maxillary artery pseudoaneurysm resulting in endovascular management of intractable bleeding.

## 2. Case Presentation

**Figure 1:**
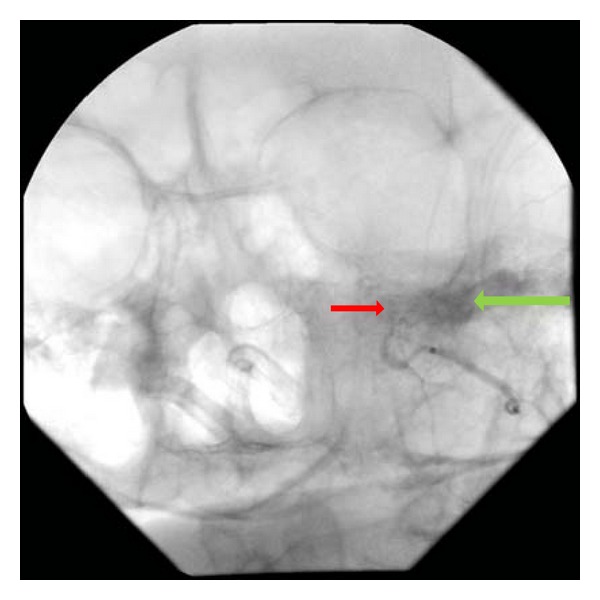
Active extravasation with filling of a small pseudoaneurysm is seen from the left internal maxillary artery.

An 86-year-old female presented to the trauma bay following a fall from standing in which she struck her face. There was no loss of consciousness, and she was not amnestic to the event. She had intractable epistaxis from both nostrils and was intubated on arrival for airway protection. On physical examination, she had profuse bleeding from both nasal passages and blood accumulating in her oropharynx. The nasal passages were packed anteriorly and posteriorly, but she continued to have bright red blood accumulate in her oropharynx. The patient was taken urgently to the angiography suite for evaluation (Figure 1). 

## 3. Discussion

Intractable epistaxis is a common reason for patients to seek emergency medical care. The majority of cases can be treated by emergency room personnel, but approximately 6% of cases are referred for Otolaryngology consultation [1]. There is a bimodal age distribution of patients with children who incur nasal trauma comprising one peak and anticoagulated elderly patients representing the other peak. Medications including nasal steroids and certain supplements including fish oil can also contribute to bleeding [2]. 

The site of bleeding is usually classified as anterior or posterior. Anterior bleeds account for 90% of all cases. They arise from Kieselbach's plexus (i.e., Little's area) located on the anteroinferior nasal septum. It represents the anastamosis of 4 arteries: anterior ethmoidal, sphenopalatine, greater palatine, and septal branch of superior labial. Because of its accessible location, anterior nasal bleeds are more easily treated with pressure, cauterization, warm water irrigation, and/or anterior nasal packing [3]. Management of anticoagulation and hypertension is usually necessary. Posterior epistaxis arises from Woodruff's plexus, a venous plexus located at the posterior aspect of the inferior meatus [4]. It can be a more challenging clinical encounter, because the site of bleeding is difficult to visualize, but most bleeds can be controlled with the application of a posterior pack and/or hemostatic agent. In some situations, bleeding is too profuse to localize the source and/or bleeding persists despite appropriate treatment. These situations warrant further investigation for a vascular injury. 

**Figure 2:**
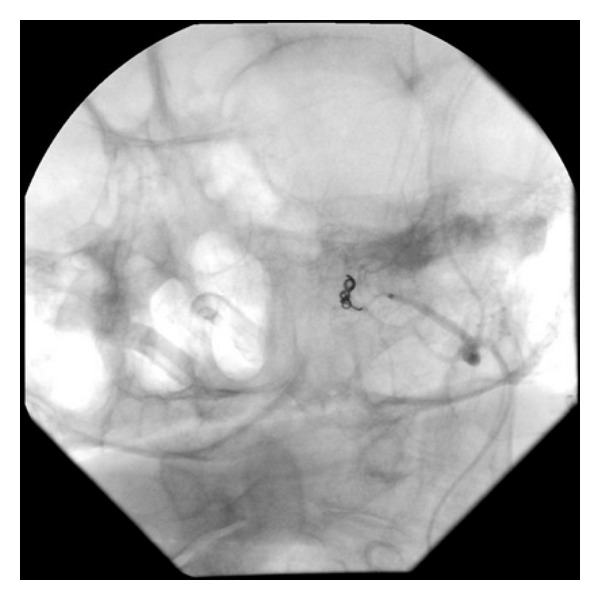
Postembolization there was minimal residual extravasation from the area supplied by the intracranial circulation (ophthalmic artery) with nonfilling of the pseudoaneurysm and complete occlusion of the left internal maxillary artery.

In our patient, a small pseudoaneurysm of the left maxillary artery with active extravasation was identified. Embolization of the left maxillary artery was performed using 500–700 micron Contour PVA followed by embolization using two 3 mm Tornado coils (Figure 2). Traumatic pseudoaneurysms are rare with an incidence of less than 1% of intracranial aneurysms [5]. The etiology includes motor vehicle accidents, pedestrians hit by cars, and falls [6]. The most common presentation is massive epistaxis months after an initial bleed and trauma [7]. In our patient, the onset of epistaxis was immediate and required intubation for airway control. It is possible that the pseudoaneurysm formed following a previous trauma, but the patient later denied any history of head trauma. Endovascular management of intractable epistaxis secondary to traumatic pseudoaneurysm has been reported in the literature as a safe and effective management option for these patients [5–8].
